# Saliva‑microbiome‑derived signatures: expected to become a potential biomarker for pulmonary nodules (MCEPN-1)

**DOI:** 10.1186/s12866-024-03280-x

**Published:** 2024-04-20

**Authors:** Yifeng Ren, Qiong Ma, Xiao Zeng, Chunxia Huang, Shiyan Tan, Xi Fu, Chuan Zheng, Fengming You, Xueke Li

**Affiliations:** 1 Hospital of Chengdu University of Traditional Chinese Medicine, Chengdu, Sichuan Province 610072 China; 2 TCM Regulating Metabolic Diseases Key Laboratory of Sichuan Province, Hospital of Chengdu University of Traditional Chinese Medicine, Chengdu, Sichuan Province 610072 China

**Keywords:** Oral microbiome, Pulmonary nodule, Liquid biopsy, Biomarkers, Clinical trial

## Abstract

**Background:**

Oral microbiota imbalance is associated with the progression of various lung diseases, including lung cancer. Pulmonary nodules (PNs) are often considered a critical stage for the early detection of lung cancer; however, the relationship between oral microbiota and PNs remains unknown.

**Methods:**

We conducted a ‘Microbiome with pulmonary nodule series study 1’ (MCEPN-1) where we compared PN patients and healthy controls (HCs), aiming to identify differences in oral microbiota characteristics and discover potential microbiota biomarkers for non-invasive, radiation-free PNs diagnosis and warning in the future. We performed 16 S rRNA amplicon sequencing on saliva samples from 173 PN patients and 40 HCs to compare the characteristics and functional changes in oral microbiota between the two groups. The random forest algorithm was used to identify PN salivary microbial markers. Biological functions and potential mechanisms of differential genes in saliva samples were preliminarily explored using the Kyoto Encyclopedia of Genes and Genomes (KEGG) and Cluster of Orthologous Groups (COG) analyses.

**Results:**

The diversity of salivary microorganisms was higher in the PN group than in the HC group. Significant differences were noted in community composition and abundance of oral microorganisms between the two groups. *Neisseria*, *Prevotella*, *Haemophilus* and *Actinomyces*, *Porphyromonas*, *Fusobacterium*, *7M7x*, *Granulicatella* and *Selenomonas* were the main differential genera between the PN and HC groups. *Fusobacterium*, *Porphyromonas*, *Parvimonas*, *Peptostreptococcus* and *Haemophilus* constituted the optimal marker sets (area under curve, AUC = 0.80), which can distinguish between patients with PNs and HCs. Further, the salivary microbiota composition was significantly correlated with age, sex, and smoking history (*P* < 0.001), but not with personal history of cancer (*P* > 0.05). Bioinformatics analysis of differential genes showed that patients with PN showed significant enrichment in protein/molecular functions related to immune deficiency and energy metabolisms, such as the cytoskeleton protein RodZ, nicotinamide adenine dinucleotide phosphate dehydrogenase (NADPH) dehydrogenase, major facilitator superfamily transporters and AraC family transcription regulators.

**Conclusions:**

Our study provides the first evidence that the salivary microbiota can serve as potential biomarkers for identifying PN. We observed a significant association between changes in the oral microbiota and PNs, indicating the potential of salivary microbiota as a new non-invasive biomarker for PNs.

**Trial registration:**

Clinical trial registration number: ChiCTR2200062140; Date of registration: 07/25/2022.

**Supplementary Information:**

The online version contains supplementary material available at 10.1186/s12866-024-03280-x.

## Background

Globally, lung cancer has one of the highest mortality rates due to its difficulty in being diagnosed early [1]. Pulmonary nodules (PNs) pose a potential risk for lung cancer. Screening for PNs can contribute to early diagnosis of lung cancer. Typically, PN is asymptomatic, and doctors often rely on imaging for the detection of PNs in the early stages of lung cancer [2, 3]. Presently, high-resolution computed tomography (CT) is the main screening method for PNs [4]; however, imaging methods alone are insufficient to accurately assess the malignancy of PNs and the prognosis of patients [5–7]. Biomarkers can be detected in the early stages of the disease, even before symptoms appear, and can be used to predict the risk, diagnosis, progression, and outcome of the disease [8, 9]. However, there is currently no research on PN biomarkers; therefore, there is an urgent need for reliable biomarkers.

The human symbiotic microbiome plays a crucial role in various biological processes and has shown enormous potential for the diagnosis and treatment of various diseases [10–13]. The oral microbiota is the second largest microbiota after the intestinal microbiota, and many studies have shown that it is associated with the occurrence and development of various disease [14, 15]. Recent research on respiratory diseases has focused on the relationship between oral microbiota and lung cancer. These studies indicate that the oral microbiota is the main source of microorganisms in the lungs and that the oral microbiota of lung cancer patients differs from that of healthy individuals. Some oral microbiota can produce sustained chronic inflammation in the oral cavity, and some strains can enter the bloodstream or directly colonize the lungs to undergo immune reactions with the host, thereby promoting the occurrence and development of lung cancer [16–20]. In view of this, some studies have utilized saliva microbiomes to examine oral microbiota imbalance and have found multiple saliva microbiota significantly associated with the risk of lung cancer. Opportunistic oral pathogens such as *Neisseria*, *Prevotella*, and *Porphyromonas* are enriched in the saliva of patients with lung cancer, and salivary microbiota imbalance is correlated with disease severity [21, 22]. These studies suggest that the characteristics of the oral microbiota can reflect disease progression and have the potential to be used as biomarkers for the non-invasive diagnosis of early lung diseases. However, most current research on oral microbiota has focused on the stage of lung cancer, and few studies have focused on the interrelationship between PNs and oral microbiota. The potential of the salivary microbiome as a biomarker for the assisted identification of PN has not been previously reported.

**Fig. 1:**
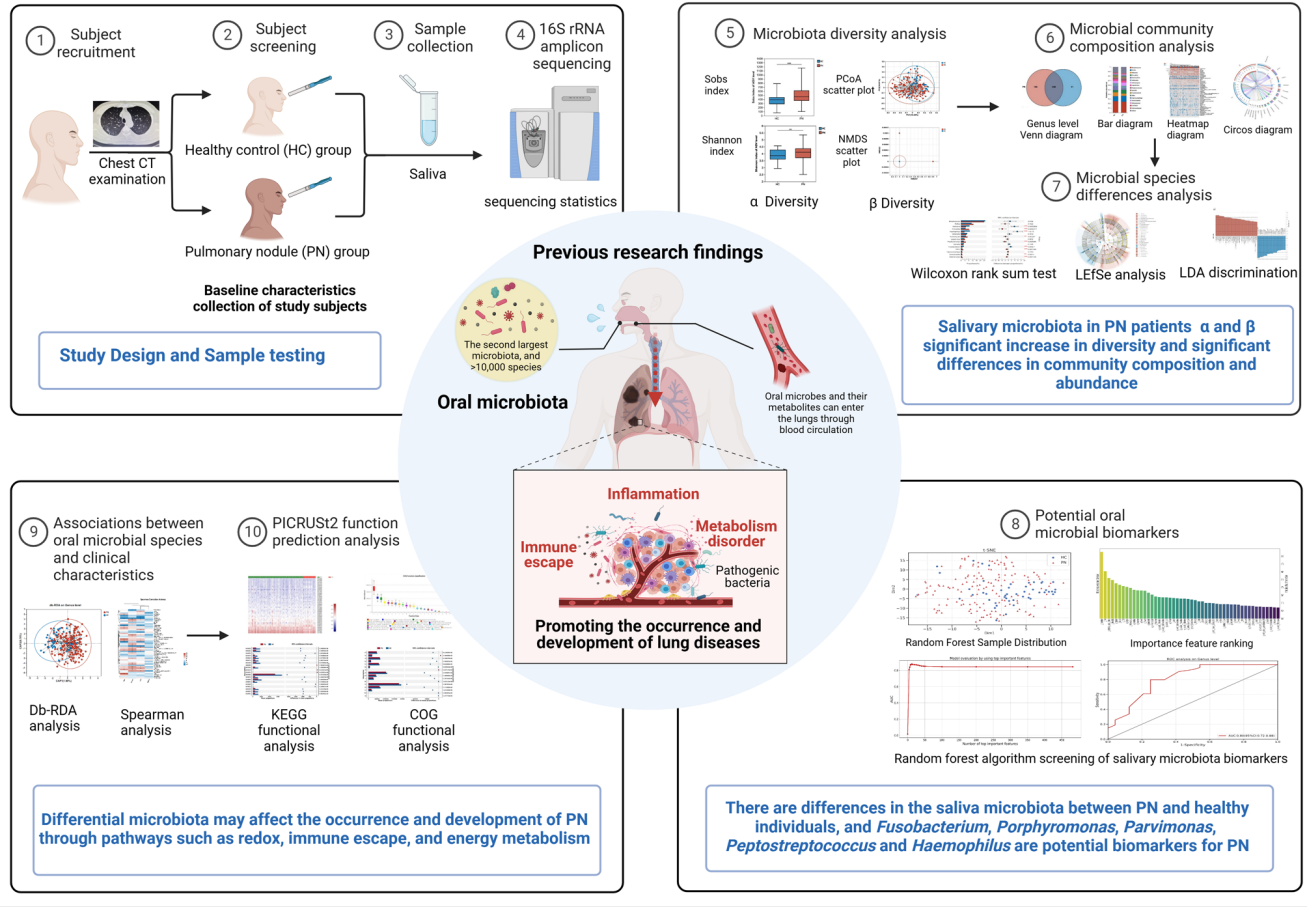
A prospective, non-randomized, concurrent controlled trial (MCEPN-1) found oral microbiome characteristics and function between healthy individuals and pulmonary nodule patients

Therefore, based on 16 S rRNA amplicon sequencing, we analyzed the characteristics and functions of the microbiota in saliva samples from patients with PNs and those from healthy individuals (MCEPN-1) (Fig. 1). We explored candidate salivary microbiota biomarkers that may have the assisted identification effects on PN and preliminarily explored how the oral microbiome affects the development of early-stage lung cancer.

## Materials and methods

### Trial design

This was a prospective, nonrandomized, concurrent controlled trial (MCEPN-1). Participants were recruited between July 2022 and March 2023 at the Hospital of Chengdu University of Traditional Chinese Medicine (TCM), Sichuan Cancer Hospital, and the Chengdu Integrated TCM and Western Medicine Hospital. This study adhered to the *Declaration of Helsinki* and was approved by the Ethics Committee of the Hospital of Chengdu University of TCM (Ethical approval Number: 2022KL-051). The trial was registered with the China Clinical Trial Registration Center (Trial Registration Number: ChiCTR2200062140; Date of registration: 07/25/2022). This study used the CONSORT reporting guidelines [23].

The following inclusion criteria applied: (1) The inclusion criteria for patients with PN is the presence of definite abnormalities on chest CT imaging that meet the diagnostic criteria for PNs according to the Fleischner Society Guidelines for Managing Incidental PNs (2017 version) [24]; the inclusion criteria for healthy control (HC) are the absence of PNs and other abnormalities on chest CT imaging; (2) voluntary consent to participate in this study obtained; (3) no personal history of cancer; (4) no history of respiratory system-related surgery; and (5) age ranging from 18 to 80 years.

The exclusion criteria were: (1) a history of untreated infectious disease; (2) a history of autoimmune disease; (3) respiratory infections, oral diseases, and other comorbidities; (4) treatment with oral antibiotics during the month before the commencement of the study; (5) undergone continuous treatment with immunosuppressants for a duration of 6 months or longer; and (6) yogurt consumption in the previous 3 days.

### Sample collection

Demographic information and clinical characteristics data were collected from each subject including: clinical data, such as sex, age, smoking history, and personal history of cancer. This information was gathered through a combination of self-reporting by the participants and extracting relevant data from their primary care notes. “Smokers” in our study referred to individuals who had a history of smoking more than 100 cigarettes throughout their lifetime. On the contrary, individuals who had not reached this threshold were categorized as “never smokers”. Before sample collection, we instructed all participants to adhere to certain restrictions: they were prohibited from dieting, smoking, and oral hygiene prophylaxis for a minimum of 3 h prior to the sampling procedure. In order to collect the oral samples in a sterile manner, participants were asked to vigorously rinse their mouths with 10 mL of sterile saline solution for a duration of 30 s [25]. CT imaging data such as the size and location of PN; and the stratified risk information for PN which was obtained by applying the Mayo Malignancy Probability Prediction Model (MPPM) recommended by the American College of Chest Physicians (ACCP). By inputting the patients’ demographic and clinical characteristics information, and imaging data including the characteristics of relevant PNs into the MPPM software, a malignancy probability score (MPS) was calculated. Based on the calculated MPS value, the MPPM categorized patients into three risk groups: the low-risk group with an MPS of less than 5%, moderate-risk group with an MPS ranging from 5 to 60%, and high-risk group with an MPS greater than 60% [26].

Oral biological samples were collected from participants as follows. The participants rinsed their mouths with drinking water before sampling, and the samplers placed a sterile EP tube on the lower lip of the participants allowing saliva to flow into the tube naturally. The non-irritating saliva was collected for 2–3 mL, preserved in dry ice, and transferred to the laboratory refrigerator set at -80 ℃ to wait for the follow-up experiment.

### Microbial DNA extraction and sequencing

We extracted total genomic DNA from the microbial community according to the manufacturer’s instructions (Omega Bio-tek, Norcr. oss, GA, Thermo Scientific). A NanoDrop2000 was used to measure DNA concentration and purity (American Thermo Scientific Company), and samples were stored at -80 ℃ for further use. Next, 16 S rRNA amplicon sequencing was performed. Using the extracted DNA as a template, the 16 S rRNA gene V3-V4 regions were amplified using universal primers 338 F (5’-ACTCCTACGGAG GCAGCAGCAGMur3’)-806R (5’-GGACTACHVGGTWTCTAAT3’) [27]. The gene products were attached with forward and reverse error-correcting barcodes in order to distinguish each sample and yield accurate phylogenetic and taxonomic information. Subsequently, PCR products were purified using NanoDrop2000 (American Thermo Scientific Company) and quantified using Quantus™ Fluorometer (Promega, USA). The normalized equimolar concentrations of each amplicon were pooled and sequenced on the MiSeq PE250 sequencing instrument (Illumina, San Diego, CA, USA). Fastp online platform (https://github.com/OpenGene/fastp, version 0.19.6) was employed to complete the quality control of the original sequence [28]. Using FLASH (http://www.cbcb.umd.edu/software/flash, version 1.2.11) software for splicing [29].

### Amplicon sequence processing and analysis

Based on default parameters, QIIME2 (version 2022.2) was used to process the biometric data of the microbiota. The DADA2 plug-in was used to sequentially filter, reduce noise, and merge and remove chimerism. The sequence after DADA2 denoising was ASVs (i.e., the variant of the amplified subsequence) [30]. Sequences from each sample were rarefied to 20,000 to minimize the effects of sequencing depth on diversity measures. Based on the Sliva 16 S rRNA gene database, the naive Bayes classifier in QIIME2 (version 2022.2) was used for species taxonomic analysis. PICRUSt2 predicted the differential community function based on ASV representative sequences. Gene family profiles were predicted, and gene pathways were identified using MinPath. The entire analysis process was performed in accordance with the PICRUSt2 protocols.

### Statistical analysis

Mothur software was used for the calculation of the α Diversity index (Sobs, Shannon) (http://www.mothur.org/wiki/Calculators) and R software (version 3.6.2) was used for visualization of the results [31]. The β diversity index is analyzed and visualized using the principal component analysis (PCoA) method based on ANOSIM/Bray Curtis algorithm and the non-metric multidimensional scale analysis (NMDS) method based on the ANOSIM/Euclidean distance metric. A Wilcoxon rank-sum test, linear discriminant analysis and the influencing factor method [Linear discriminant analysis Effect Size (LEfSe); http://huttenhower.sph.harvard.edu/LEfSe; Latent dirichlet allocation (LDA) > 3, *P* < 0.05] were used to estimate species abundance differences between groups [32]. We visualized the distribution of samples using t-Distributed Stochastic Neighbor Embedding (t-SNE) technique. Random forest models were realized using the random Forest function from the random forest R package. Feature importance scores were generated using a random forest classifier with 80% training and 20% testing sets. After the training, we predicted the testing dataset with the resulting random forest model, and combined the predictions with the testing observations. Finally, the observations that occurred during the training process of risk factors based on area under the curve (AUC) verification was used to assess disease prediction efficiency of the saliva samples. Based on the Bray-Curtis distance, the effect of clinical characteristics on salivary microbial community composition was evaluated using distance-based redundancy analysis (db-RDA). The correlation between the salivary microbial community composition and clinical characteristics of the subjects was assessed using Spearman’s correlation coefficient, and the results were visualized through a heatmap diagram. The R package MaAsLin2 (Microbiome Multivariable Associations with Linear Models) from MaAsLin2 framework (adjusted for age, gender, smoking history and personal history of cancer; PN was set as reference group) was used to determine multivariable associations between microbial taxa (the relative abundance of bacterial results was used) and PN/ HC. It was considered statistically robust if the Spearman’s correlation coefficient was over 0.6 or less to -0.6, with a *P*-value less than 0.001. In addition, we used the Wilcoxon rank sum test to compare the differences in demographic and clinical characteristics between groups. All the methods were performed in accordance with relevant guidelines and regulations.

## Results

### Clinical characteristics of study subjects

**Fig. 2:**
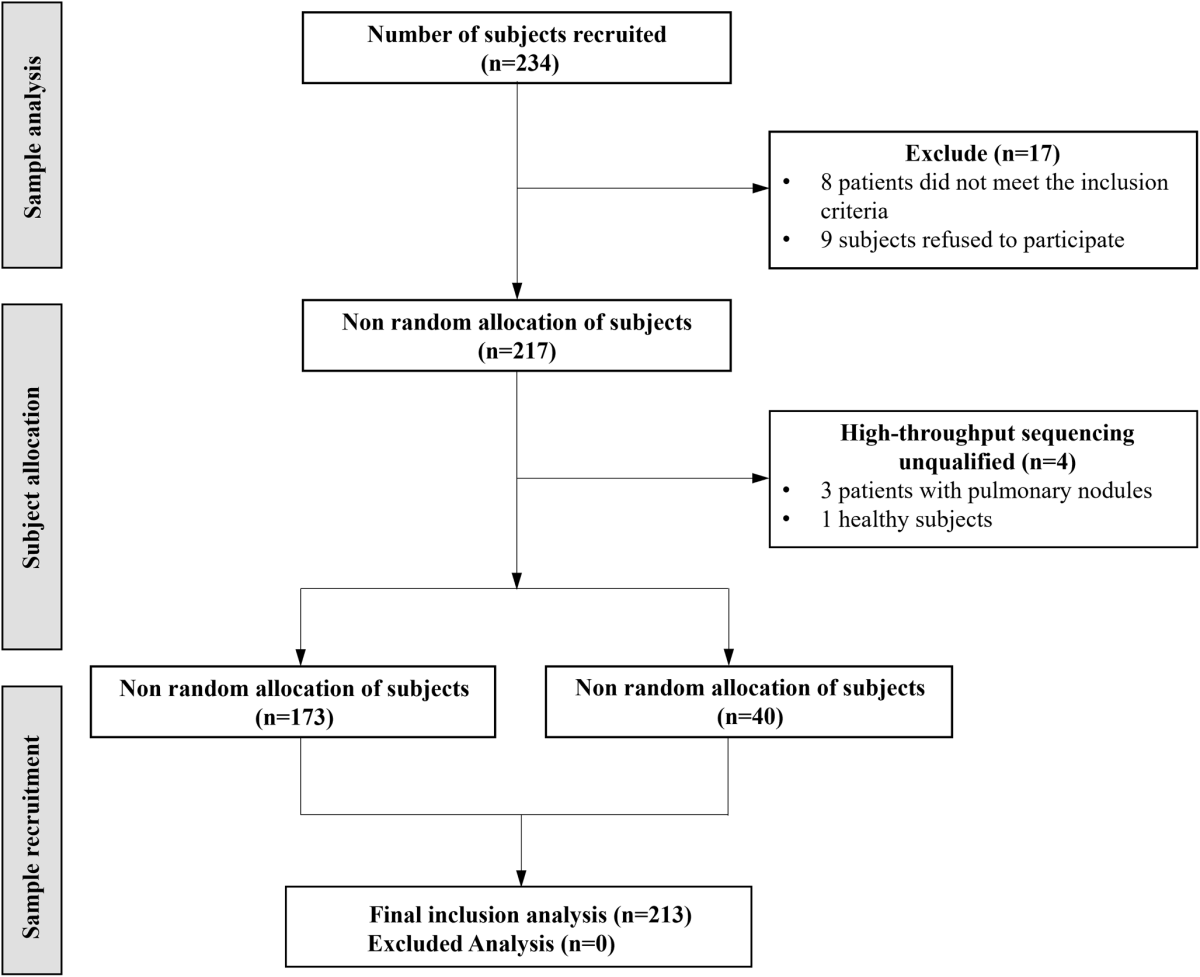
Recruitment profile of MCEPN-1 study

This study recruited 234 subjects. Following the exclusion of 17 subjects who did not meet the inclusion criteria or refused to participate, a total of 217 subjects underwent a non-random assignment. Four subjects were subsequently excluded based on unqualified 16 S rRNA amplifying results. Saliva samples from 173 patients with PNs and 40 healthy individuals were included in the final analysis (Fig. 2). The comparison of demographic and clinical characteristics between the two groups revealed that, except for smoking history, baseline characteristics, such as age and gender, did not differ significantly between the groups (Table 1).

**Table 1:** Patient characteristics

Characteristics	PN group (n = 173)	HC group (n = 40)	*P* value
**Age (years), median (IQR)** ^**a**^	45 (32, 55)	42 (33, 48)	0.39
**Gender, n (%)** ^**b**^			0.25
Male	69 (39.9%)	12 (30%)	
Female	104 (60.1%)	28 (70%)	
**Smoking history, n (%)** ^**b**^			< 0.001
NO	134 (77.5%)	40 (100.0%)	
Yes	39 (22.5%)	0 (0.0%)	
**Personal history of cancer, n (%)** ^**c**^			0.99
NO	167 (96.5%)	39 (97.5%)	
Yes	6 (3.5%)	1 (2.5%)	
**Risk stratification of PN, n (%)**			
Unable to layer	9 (5.2%)	/	
Low risk	100 (57.8%)	/	
Moderate risk	58 (33.5%)	/	
High risk	6 (3.5%)	/	

*P* < 0.05 was considered a statistically significant difference

IQR, interquartile range; PN, pulmonary nodule; HC, healthy control

a: Wilcoxon rank sum test; b: Chisq test; c: Yates’ correction

### Oral microbiota profile alterations in PN patients

#### Sample overview

After sequence denoising, pruning, and chimera filtering, we obtained a total of 19,236,964 optimized sequences and 8,117,947,662 bases from the saliva samples, with an average sequence length of 422 bp. Species annotations included one domain, one kingdom, 24 phyla, 56 classes, 124 orders, 222 families, 482 genera, 1047 species and 40,555 ASVs. The pan/core species analysis showed that the sample size was relatively sufficient (Supplementary Fig. 1A, B), and the rank-abundance curve showed that the saliva samples had high microbial species richness and evenness (Supplementary Fig. 2).

#### Biodiversity between PN patients and healthy individuals

**Fig. 3:**
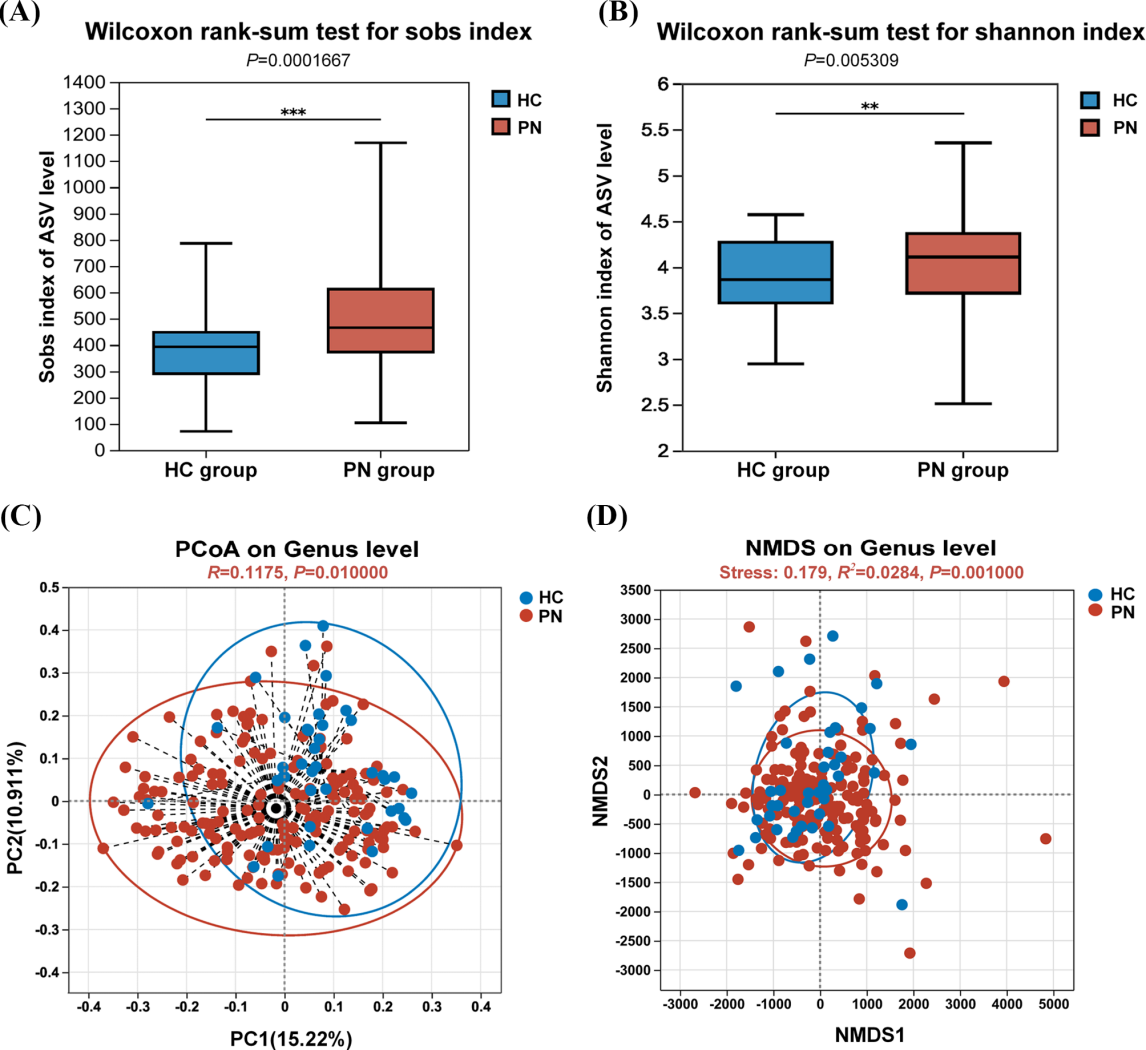
Analysis of the α- and β- diversity indices between PN group and HC group. (**A**) Comparison of Sobs index between PN group and HC group. Wilcoxon rank sum test, *P* = 0.0001667). (**B**) Comparison of Shannon index between PN group and HC group. Wilcoxon rank sum test, *P* = 0.005309). (**C**) Scatter plot of the comparison of β diversity analysis conducted with PCoA between PN group and HC group. Samples from the PN group and HC group are tightly clustered and separated from each other on the plot, it suggests that there are distinct microbial community differences between the two groups. (**D**) Scatter plot of the comparison of β diversity analysis conducted with NMDS between PN group and HC group. The plot represents the distribution and clustering patterns of samples based on their microbial composition. Each point represents a sample, and the position of the points reflects the dissimilarity in microbial composition between samples. A larger distance indicates a greater dissimilarity, while a shorter distance suggests a higher similarity in microbial composition

Next, we compared the oral microbiota diversity of patients with PN to that of healthy individuals. α diversity describes the species richness and species diversity in the microbial community by calculating the Sobs index and Shannon index. The dilution curve analysis of the Sobs and Shannon indices showed that the dilution curve was smooth and flat, indicating that the sequencing depth was sufficient, and the sequencing data was reasonable, reflecting the microbial diversity information of most samples (Supplementary Fig. 3A, B). Compared with the HC group, the Sobs index (496.11 *v.s*. 378.95, *P* < 0.001) (Fig. 3A) and Shannon index (6.05 *v.s*. 3.89, *P* = 0.005) (Fig. 3B) in the PN group were significantly higher than in the healthy control group (HC), indicating that the α diversity of the oral microbiota in patients with PN was higher than that in healthy people.

β diversity performance evaluates the species diversity of the overall microbiota between the two communities. We first used sample hierarchical cluster analysis to show that there may be differences in oral microbiota similarities between patients with PN and healthy individuals (Supplementary Fig. 4A, B, C). Subsequently, we used the PCoA analysis method based on the ANOSIM/ Bray Curtis algorithm and the NMDS analysis method based on the ANOSIM/Euclidean distance metric to analyze the salivary samples of two groups using β diversity testing of inter-group differences for diversity (Fig. 3C, D), to determine whether the species diversity could distinguish the PN group from the HC group. The results showed that, compared to the intra-group difference, the difference in saliva sample microbiota composition between the PN and HC groups was more significant (*R* = 0.118, *P* = 0.01; *R*^*2*^ = 0.028, *P* = 0.001). This was consistent with the results of the sample hierarchical cluster analysis, indicating that saliva microbiota were significantly different between PN patients and healthy subjects. These results suggested that the oral microbiota of PN patients is ecologically unbalanced.

#### Composition of microbiota communities in PN patients and healthy subjects

**Fig. 4:**
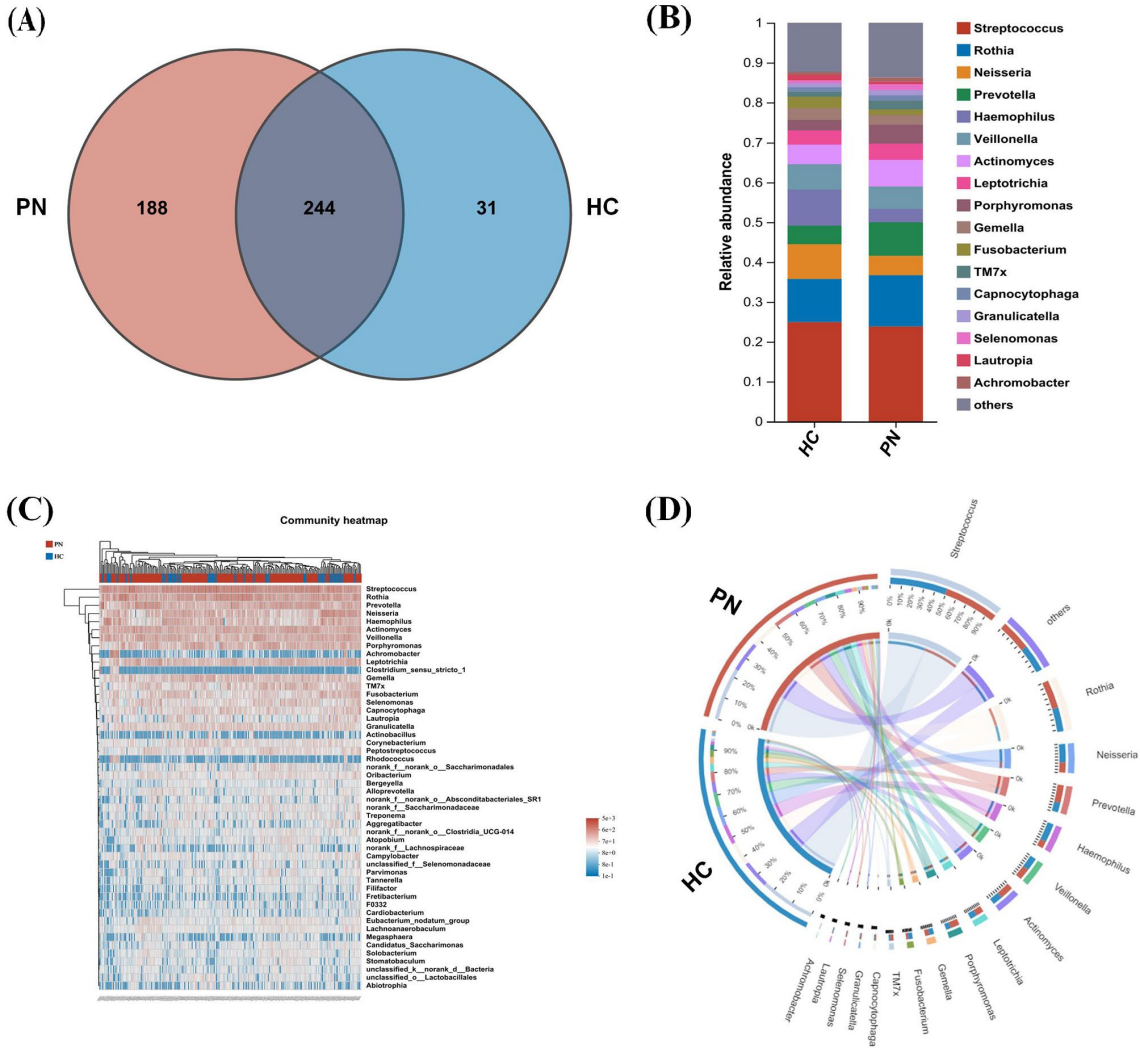
(**A**) Venn diagram of genera between PN group and HC group. The shared region represents genera that are present in both groups, while the separate regions represent genera specific to each group. The size of each region corresponds to the number of genera within it. (**B**) Relative abundance (%) of genera in the PN and HC groups. Genera are arranged on the y-axis, and the x-axis represents the relative abundance (%) of each genus. The different colors represent different genera, and the heights of the bars indicate the abundance of each genus. (**C**) Heatmap of the salivary sample similarity and difference matrix. Each row and column in the heatmap correspond to a sample, and the color intensity reflects the degree of similarity or difference between samples. (**D**) The co-occurrence relationships of core bacteria between the PN and HC groups. Each node represents a bacterial genus, and the edges indicate co-occurrence relationships between them. The size of the nodes reflects the abundance or prevalence of each bacterial genus, while the thickness of the edges represents the strength of their co-occurrence relationship

The Venn diagram at the genus level (Fig. 4A) showed 188 unique genus in the PN group and 31 unique genus in the HC group, with 244 genus in both groups. To further analyze the differences in the genus level microbiota between PN patients and healthy subjects, we compared the relative abundance of microbiota composition between the PN group and HC group using a community bar map, heatmap map, and Circos map (Fig. 4B, C, D). The top 5 genera with relative abundance in the PN group was: *Streptococcus* (23.86%), *Rothia* (12.83%), *Prevotella* (8.40%), *Actinomyces* (6.65%) and *Veillonella* (5.59%); while the top 5 bacteria in HC group were: *Streptococcus* (24.94%), *Rothia* (10.82%), *Haemophilus* (9.06%), *Neisseria* (8.72%), and *Veillonella* (6.33%). The results show differences in the most abundant taxa between PN and HC groups.

#### Microbiota differences at genus level between patients with PN and healthy subjects

**Fig. 5:**
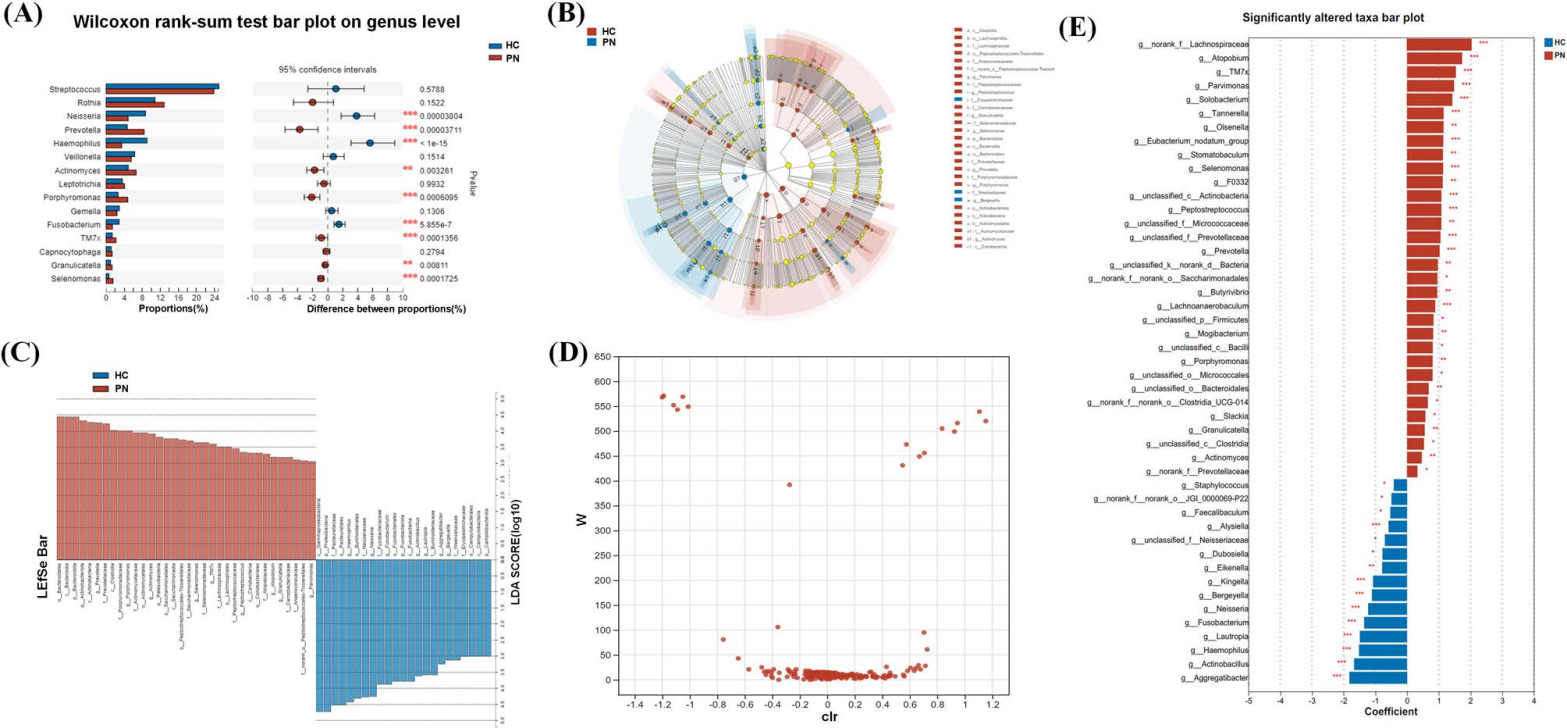
(**A**)Wilcoxon rank-sum test bar plot on genus level between the PN and HC groups. Each bar represents a genus, and the height of the bar indicates the magnitude of the difference in abundance between the PN and HC groups. (**B**) Cladogram plot of LEfSe analysis indicating the enriched taxa of saliva microbiome in PN and HC groups. The central point represents the root of the tree (Bacteria), and each ring represents the next lower taxonomic level (phylum to genus: p, phylum; c, class; o, order; f, family; g, genus). The diameter of each circle represents the relative abundance of the taxon. (**C**) Histogram of linear discriminant analysis (LDA) scores (> 3) for differentially abundant genera between two groups. Each bar represents a genus, and the height of the bar indicates the strength of its discriminatory power. The cutoff of LDA score > 3 indicates significant differential abundance between the two groups. (**D**) The volcano plot of the comparison of differences in abundance of common genera by analysis of the composition of microbiomes (ANCOM) analysis. Each scatter in the diagram represents a compared genera. Each scatter on the plot represents a compared genus, with the ordinate representing the W value and the abscissa representing the CLR (Center Log Transform). The CLR represents the degree of difference in sample abundance between groups, with larger absolute values indicating greater relative abundance differences. (**E**) The association between PN and HC groups with oral microbiome features as determined by MaAsLin2 (adjusted for age, gender, smoking history and personal history of cancer; PN was set as reference group) (*P* < 0.05). Each bar represents a specific bacterial taxon, and the height of the bar represents the magnitude of enrichment or depletion in either the PN or HC group. * 0.01 < *P*-value ≤ 0.05, ** 0.001 < *P*-value/≤ 0.01, *** *P*-value ≤ 0.001

The Wilcoxon rank-sum test and LEfSe analysis were used to confirm the genus differences and identify potential oral microbial biomarkers. Compared with the HC group (Fig. 5A, B, C), there were nine genera with significant differences between the PN and HC groups (*P* < 0.05), including *Neisseria*, *Prevotella*, *Haemophilus*, *Actinomyces*, *Porphyromonas*, *Fusobacterium*, *7M7x*, *Granulicatella* and *Selenomonas*. The saliva samples showed a rich diversity of genus according to the ANCOM difference test results at the genus level (Fig. 5D). Furthermore, we utilized MaAsLin2 to examine the association between the oral microbiome and clinical features of PN. Compared with the HC group, the baseline oral microbiota composition of PN was enriched in 19 bacterial genus and characterized by the genera including *Porphyromonas*, *Lachnoanaerobaculum*, *Prevotella* and *7M7x* (Fig. 5E).

### Potential microbe biomarkers for PN

**Fig. 6:**
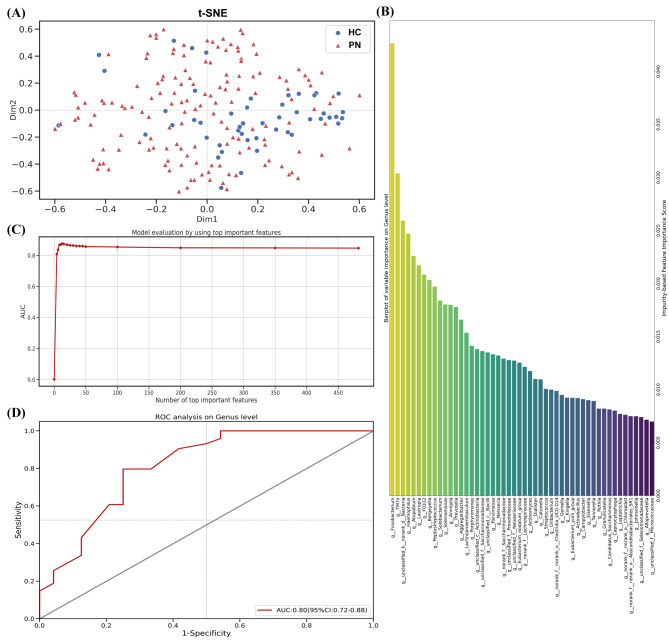
(**A**) Two-dimensional scatter plot of saliva sample based on t-Distributed Stochastic Neighbor Embedding (t-SNE) technique. (**B**) Bar plot of species importance at the genus level performed by random forest algorithm. Each bar represents a genus, and the height of the bar indicates the importance or contribution of that genus to the overall predictive power of the random forest model. Genera with higher importance values have a stronger influence on the classification outcome. (**C**) Trend graph of area under the curve (AUC) increasing with the number of top important features. (**D**) Receiver operating characteristic (ROC) curve of saliva sample for predicting pulmonary nodules at the genus level. The x-axis of the curve represents the false positive rate (1-specificity), and the y-axis represents the true positive rate (sensitivity)

To further identify the characteristic oral microbial genera related to PN and evaluate the potential of salivary biological samples as new non-invasive biomarkers, we discriminated the disease prediction efficiency of salivary microbiota using a random forest model (Fig. 6). As shown in Fig. 6A, the distribution of microbial communities in the two groups of saliva samples is uniform. Subsequently, we selected the genus with the top 15 importance features and the accuracy of the validation AUC was 87.00% (Fig. 6B, C). After excluding 11 unclassifiable and less clinically relevant genera from the literature, *Fusobacterium*, *Porphyromonas*, *Parvimonas*, *Peptostreptococcus* and *Haemophilus* formed the optimal biomarker sets. By utilizing only these five features, the AUC value can reach 0.80 (95% CI: 0.72–0.88) (Fig. 6D). This prediction model identified five microbial genera that could be used to distinguish between patients with PN and healthy populations.

### Correlation between the oral microbiota and clinical characteristics of PN

**Fig. 7:**
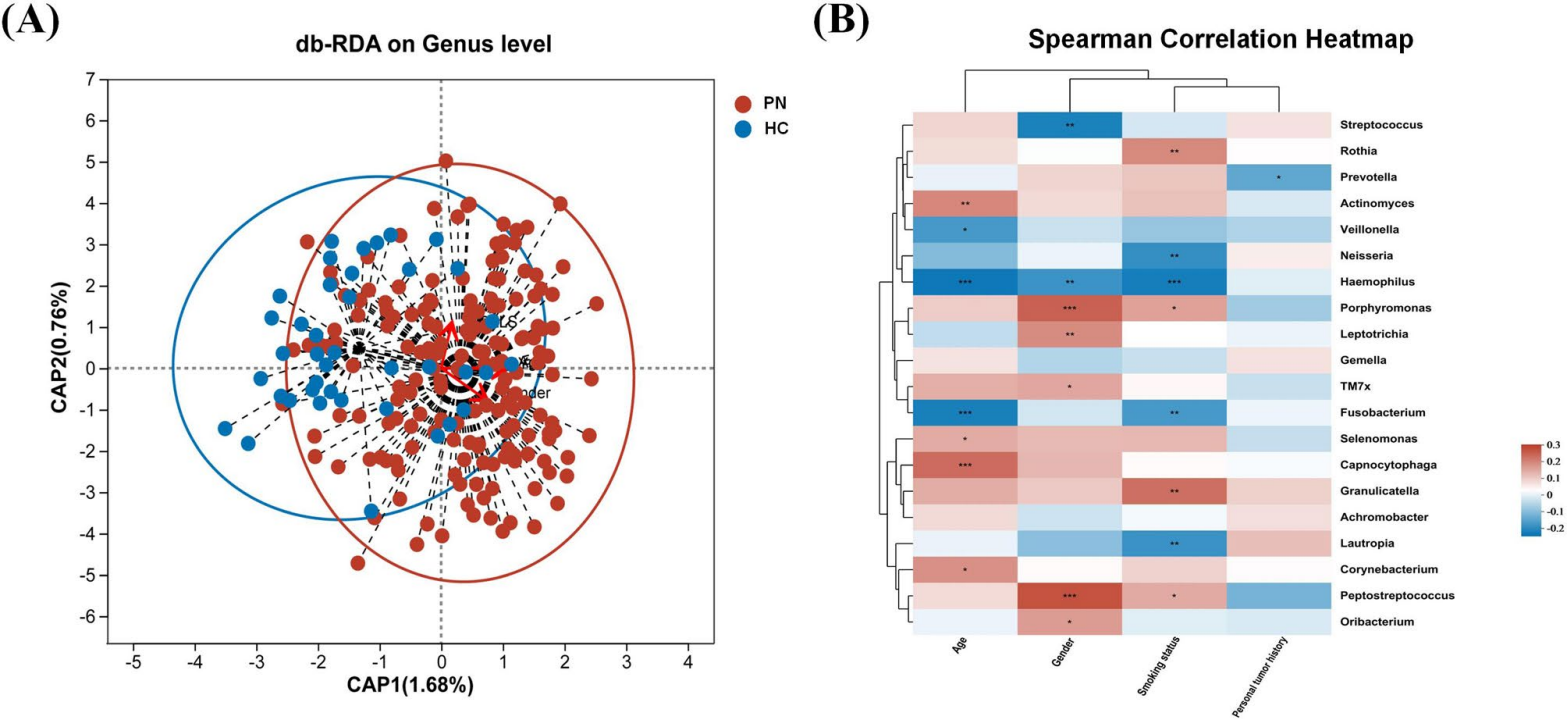
db-RDA analysis based on Bray-Curtis distance between environmental factors and bacterial groups (genus level). Each point on the plot represents a sample point. The arrows indicate the direction and magnitude of the influence of each environmental factor on the bacterial genera, and the length and direction of the arrow represent the strength and direction of the correlation. (**B**) Heatmap diagram based on Spearman correlation analysis between environmental factors and bacterial groups (genus level). Each row corresponds to an environmental factor, and each column represents a bacterial genus. The colors in the heatmap represent the Spearman correlation coefficients

In addition, we used db-RDA (based on Bray-Curtis distance) to evaluate the correlation between the clinical characteristics of the subjects and the composition of the salivary microbiota. Before the correlation analysis, we identified environmental factors affecting microbial communities by using the variance inflation factor (VIF < 5). The VIF values of the subjects’ clinical characteristic environmental factors did not change before and after identification, which could be used for the follow-up analysis (see Supplementary Table 1). The subsequent db-RDA analysis results showed that all sample points formed two clusters, indicating that clinical features have a potential impact on the composition of salivary microbiota in patients with PN (Fig. 7A).

The Spearman correlation coefficient was used to evaluate the correlation between the composition of the salivary microbiota and the clinical characteristics of the subjects (Fig. 7B). In general, age, gender, and smoking history were positively or negatively correlated with salivary microbiota (*P* < 0.001, *r* > 0.6/ *r*< -0.6). Age was positively correlated with *Capnocytophaga* (*r* = 0.228, *P* < 0.001) and negatively correlated with *Fusobacterium* (*r*= -0.225, *P* < 0.001) and *Haemophilus* (*r*= -0.237, *P* < 0.001). Gender was positively correlated with *Peptostreptococcus* (*r* = 0.267, *P* < 0.001) and *Porphyromonas* (*r* = 0.245, *P* < 0.001), negatively correlated with *Streptococcus* (*r*= -0.221, *P* = 0.001). Smoking history was positively correlated with *Granulicatella* (*r* = 0.223, *P* = 0.001), negatively correlated with *Haemophilus* (*r* = -0.227, *P* < 0.001), and had no significant association with personal history of cancer (Supplementary Table 2). These results suggest that changes in salivary microbiota combined with host age and smoking history may affect the disease process.

### Functional potentials of the oral microbiome associated with PN

**Fig. 8:**
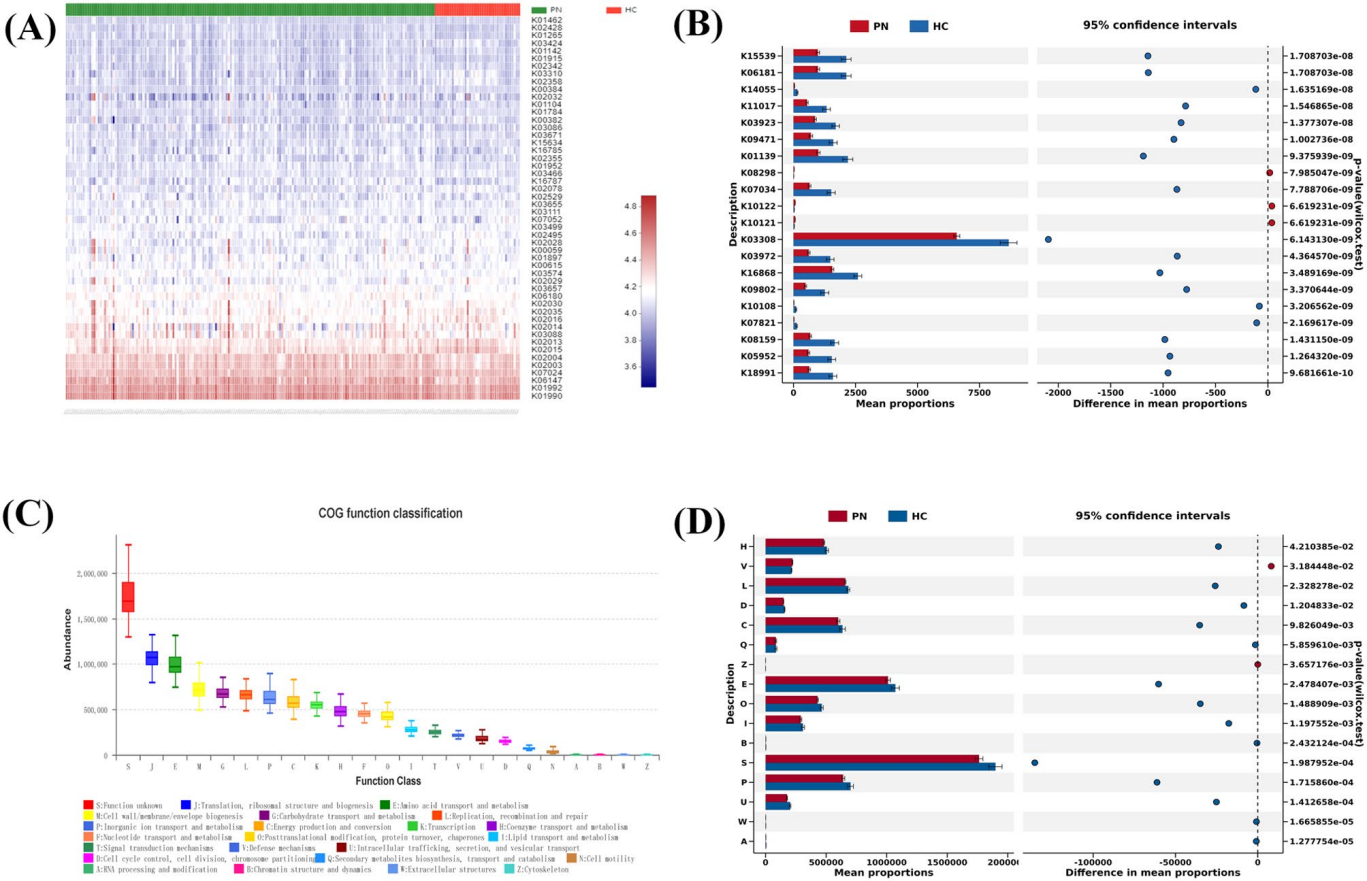
Functional profiling performed with PICRUSt2. (**A**) Q-value heatmap of KEGG function enrichment in two groups. The colors in the heatmap indicate the q-value, which is a measure of false discovery rate (FDR) adjusted p-value. Lighter colors indicate lower q-values, indicating higher significance or enrichment of the KEGG function in that group. (**B**) Histogram of KEGG pathway enrichment analysis between PN and HC groups. Histogram of KEGG pathway enrichment analysis between PN and HC groups. (**C**) Box-plot of COG functional classification. Each box in the plot represents a specific COG functional category, and the height of the box indicates the range or dispersion of the category’s abundance or frequency across the samples or groups. (**D**) Histogram of COG pathway enrichment analysis between PN and HC groups. Each bar in the histogram represents a COG pathway, and the height of the bar indicates the enrichment score or significance level. Higher bars indicate more significant enrichment in the corresponding COG pathway

We examined the functional changes in the oral microbiome of patients with PN to understand the relationship between the oral microbiome and PN development. The Kyoto Encyclopedia of Genes and Genomes (KEGG) orthology (KO) function abundance results showed that the oral microbiome in the PN group was enriched in cytoskeleton protein RodZ, pseudouridine 2457 synthase, universal stress protein E, hemolysin activated/secreted protein, nicotinamide adenine dinucleotide phosphate (NADPH) dehydrogenase γ- Functional genes related to glutamyl putrescine oxidase, Na^+^ transporter, phage shock protein E, tellurium methyltransferase, MFS transporter protein, and AraC family transcription regulatory factor (Fig. 8A, B). The COG Function classification analysis showed that in the PN group, the abundance of functional genes related to RNA processing and modification, chromatin structure and dynamics, energy generation and conversion, cell cycle control, cell division, chromosome division, amino acid transport, and metabolism were significantly reduced (Fig. 8C, D). These data suggest that the oral microbiota may participate in the process of PN disease through pathways such as redox reactions, immune escape, and energy metabolism.

## Discussion

The oral microbiota is rich in species and comprises large quantities of bacteria. The continuity of the microbiome from the oral cavity to the lower respiratory tract makes the oral microbiota the main determinant of the lung microbiota with important potential for clinical application, in the early diagnosis and treatment of lung diseases. A recent study found that using saliva samples for detection has a higher sensitivity and specificity than using nasal and pharyngeal swabs [33]. Similarly, Professor Ding’s team observed the shaping process of the lung microbiota using multi-source microbiota, such as saliva, nasal cavity, oropharynx, and bronchoalveolar lavage fluid samples, as well as the systematic correlation between oral microbiota and lung microbiota [34]. The results also confirmed the enormous value of saliva samples for evaluating lung microbiota. The results of these studies suggest that changes in the salivary microbiome can serve as non-invasive biomarker for pulmonary microbiome ecological imbalance or lung pathogen invasion. However, no studies have been conducted on the microbial characteristics or diagnostic efficacy of saliva samples obtained from PN patients [35]. MCEPN-1 is the first research to conduct a prospective, multicenter, non-randomized, concurrent controlled trial to determine the diagnostic efficacy of oral microbiome markers.

Microbiome diversity is an important parameter for characterizing microbial communities. Growing evidence suggests that changes in microbiome diversity are associated with disease risk [36]. We found that the salivary microbiota α and β diversity index of patients with PN was higher than that of HCs, indicating that there was an ecological imbalance in the oral microbiota of patients with PN. It is worth mentioning that a related discovery was that primary or recurrent lung cancer had a more α diversity microbiota than healthy lung tissue [37]. Additionally, Zeng et al. discovered that the microbiota in the lungs show increased α diversity and significant changes in β diversity during carcinogenesis [38]. The results were consistent with the results of the α and β diversity index in the present study. However, Shi et al. [39] and Tsay et al. [16] found no differences in diversity between lung cancer patients and healthy subjects. It is possible that the discrepancy between our results is due to differences in the living environment, the number of samples collected, the control group, or the way the sequencing data was analyzed.

In this study, we identified the microbiota characteristics and differences in the saliva of patients with PN for the first time. The saliva samples from patients with PN was significantly enriched in *Prevotella*, *Porphyromonas*, *Actinomyces*, *Selenomonas*, *Granulicatella* and *7M7x*. This was corroborated by using the Wilcoxon rank-sum test and LEfSe analysis, demonstrating the stability and reliability of our statistical analysis. To date, no studies have reported evidence of microorganisms and PN for horizontal comparison. However, the potential of microbes as novel biomarkers for distinguishing patients with lung cancer from healthy individuals and patients with benign lung lesions has been previously suggested. Zhang et al. reported that compared with healthy subjects, the number of *Veillonella* and *Streptococcus* in patients with NSCLC increased, while *Fusobacterium* and *Prevotella* decreased [40]. Kovaleva et al. reported similar results for NSCLC specimens [41]. Another study found that the abundances of *Veillonella* and *Capnocytophaga* in the saliva of patients with lung cancer were higher than those in healthy individuals. Importantly, the enrichment characteristics of *Veillonella* and *Capnocytophaga* in saliva can distinguish healthy individuals from patients with lung cancer [42]. These results suggest that lung cancer may be related to an imbalance in the salivary microbiome, which has diagnostic value for lung cancer. Although there are differences between these studies, these show that oral pathogenic bacteria potentially play an important role in the microbial environment during the development of lung cancer. To a certain extent, oral microbial markers may have diagnostic value in the evolution of lung nodules and lung cancer. Oral microbial markers may have potential diagnostic value in the development of PN and lung cancer. In addition, to more accurately evaluate the potential of saliva as a new non-invasive biomarker, we conducted a discriminant analysis on disease prediction efficacy of saliva samples based on the random forest model verified by the AUC. We identified five microbial genera, *Fusobacterium*, *Porphyromonas*, *Parvimonas*, *Peptostreptococcus* and *Haemophilus* as the best predictors, which can distinguish patients with PN from HCs. Therefore, oral microbes may contribute to the assisted identification of PN; however, the exact underlying mechanism remains to be explored.

According to previous reports, clinical characteristics such as sex, age, and smoking history can affect the severity of PN and lung cancer [43, 44]. An imbalance in microbiome ecology can promote an environment conducive to the development of PN disease, thus possessing the potential to become a risk factor for disease severity. This study examined whether salivary microorganisms are positively or negatively correlated with PN characteristics. These results suggest that changes in the oropharyngeal microbiota may affect the occurrence and development of PN due to their interaction with age, smoking history, and sex indices in patients with PN. Therefore, oral microbes are expected to be reliable indicators of PN severity.

In addition, we preliminarily explored the bioinformatics function of differential genes in saliva samples from patients with PN and healthy individuals using PICRUSt2 analysis based on the KEGG and COG databases. We found that the cytoskeletal proteins RodZ, NADPH dehydrogenase, oligofructose transport system osmotic enzyme protein, and other proteins/molecular functions directly related to energy metabolism were the most significantly enriched, and their abundance showed varying degrees of change. A similar conclusion was reached regarding the statistical results of COG Function classification. Recent studies have reported that salivary microbiota can affect the p53 and apoptosis signaling pathways in lung cancer cells [45]. Additionally, salivary microbiota have been shown to influence systemic inflammation in patients with cancer [46]. *Veillonella* in the saliva of patients with NSCLC is positively correlated with the neutrophil-to-lymphocyte ratio, whereas *Streptococcus* is negatively correlated with the lymphocyte-to-monocyte ratio [47]. Yu et al. found that cathepsin downregulation, peroxidase activity, and cell redox homeostasis were significantly downregulated in a lung cancer group [48]. Cellular redox homeostasis is a key indicator for maintaining a symbiotic relationship between the microbiota and host [49]. Dysregulation of this function can lead to microbiome dysbiosis and inflammation within the host, which can lead to lung cancer and PN [50, 51]. The above experimental evidence is consistent with the results of the bioinformatics analysis in this study. Therefore, the downregulation of microbiota abundance and the corresponding redox function may be an important starting point for revealing redox homeostasis imbalances in PN in the future. Although long-term immune responses are associated with chronic inflammation and carcinogenesis, increasing evidence suggests that microbiomes shape adaptive immunity to evade immune surveillance [52, 53]. Research has found that in lung cancer, there is a significant enrichment in flagellar assembly pathways related to energy metabolism, an increase in bacterial migration ability and cell development level, and a decrease in immune-related functions [54]. Therefore, immune deficiency and abnormal energy metabolism may lead to the occurrence of PN or “nodule-cancer” transformation driven by microbial translocation. In summary, the current limited research provides new evidence and interpretable evidence for the mechanism of oral microbiota in the occurrence and development of PN.

Our study is an initial exploration of the relationship between PN and oral microbiota. However, there are several limitations to our study. First, there is no classification of benign and malignant PNs, which makes the results not necessarily representative, and our research group will further explore this in subsequent follow-up studies. Second, there is lack of mechanistic research in cell and animal experiments to confirm whether the discovered microbial differences can be used as biomarkers.

## Conclusions

In this study, we observed a significant association between changes in oral microbiota and PN. These findings suggest a potential link between oral microbiota and PN. Potential molecular markers for PN include *Fusobacterium*, *Porphyromonas*, *Parvimonas*, *Peptostreptococcus* and *Haemophilus*. In addition, changes in oral microecology may be associated with the development of PN as a result of induction of host immune deficiency and abnormal cellular redox homeostasis. Future prospective studies with a follow-up period and incident PN cases are needed to validate the potential of salivary microbiota as a non-invasive biomarker for early detection and prediction of PN.

### Electronic supplementary material

Below is the link to the electronic supplementary material.

Supplementary Material 1

Supplementary Material 2

Supplementary Material 3

Supplementary Material 4

Supplementary Material 5

Supplementary Material 6

## Data Availability

All raw 16 S rRNA gene sequencing data have been deposited in the NCBI Sequence Read Archive (SRA) under accession number SUB13190793 (BioProject: PRJNA979793).

## Abbreviations

MCEPN-1: Microbiome with pulmonary nodule series study 1
PN: Pulmonary nodule
HC: Healthy control
TCM: Traditional Chinese medicine
KEGG: Kyoto encyclopedia of genes and genomes
KO: Kyoto encyclopedia of genes and genomes orthology
AUC: Area under curve
CT: Computed tomography
ACCP: American college of chest physicians
PCoA: Principal component analysis
NMDS: Non-metric multidimensional scale
LEfSe: Linear discriminant analysis effect size
NSCLC: Non-small cell lung cancer
IQR: Interquartile range
