# Supplementary material for: Saliva‑microbiome‑derived signatures: expected to become a potential biomarker for pulmonary nodules (MCEPN-1)
Source: BMC Microbiol. 2024 Apr 20;24:132. doi: 10.1186/s12866-024-03280-x (PMC11031921; doi:10.1186/s12866-024-03280-x)
Supplement: Supplementary file 1 — Supplementary Material 1 [file 12866_2024_3280_MOESM1_ESM.docx]

| **Supplementary Table 1** VIF variance inflation factor collinearity analysis | | | | |
| --- | --- | --- | --- | --- |
| **Factor** | **VIF value** | | **Factor** | **VIF value** |
| **Environmental factor VIF value before screening** | | | | |
| Age | 1.08610272033186 | Gender | | 1.36666770970373 |
| Smoking history | 1.37991739233775 | Personal history of cancer | | 1.01563553235591 |
| **Environmental factor VIF value after screening** | | | | |
| Age | 1.08610272033186 | Gender | | 1.36666770970373 |
| Smoking history | 1.37991739233775 | Personal history of cancer | | 1.01563553235591 |
