# Supplementary material for: Saliva‑microbiome‑derived signatures: expected to become a potential biomarker for pulmonary nodules (MCEPN-1)
Source: BMC Microbiol. 2024 Apr 20;24:132. doi: 10.1186/s12866-024-03280-x (PMC11031921; doi:10.1186/s12866-024-03280-x)
Supplement: Supplementary file 2 — Supplementary Material 2 [file 12866_2024_3280_MOESM2_ESM.docx]

| **Supplementary Table 2** db-RDA Environmental factor analysis | | | | |
| --- | --- | --- | --- | --- |
| **Characteristic factor** | **CAP1** | **CAP2** | **R^2^** | ***P*** |
| Age (years; mean±SD) | 0.9998 | 0.0177 | 0.1876 | 0.001 |
| Sex (M/F) | 0.66901 | -0.7238 | 0.1150 | 0.001 |
| Smoking status (Y/N) | 0.9998 | -0.0200 | 0.1430 | 0.001 |
| Personal history of cancer | -0.9356 | 0.9877 | 0.0210 | 0.124 |
