# Supplementary material for: Saliva‑microbiome‑derived signatures: expected to become a potential biomarker for pulmonary nodules (MCEPN-1)
Source: BMC Microbiol. 2024 Apr 20;24:132. doi: 10.1186/s12866-024-03280-x (PMC11031921; doi:10.1186/s12866-024-03280-x)
Supplement: Supplementary file 3 — Supplementary Material 3 [file 12866_2024_3280_MOESM3_ESM.docx]

**Supplementary Figure 1**


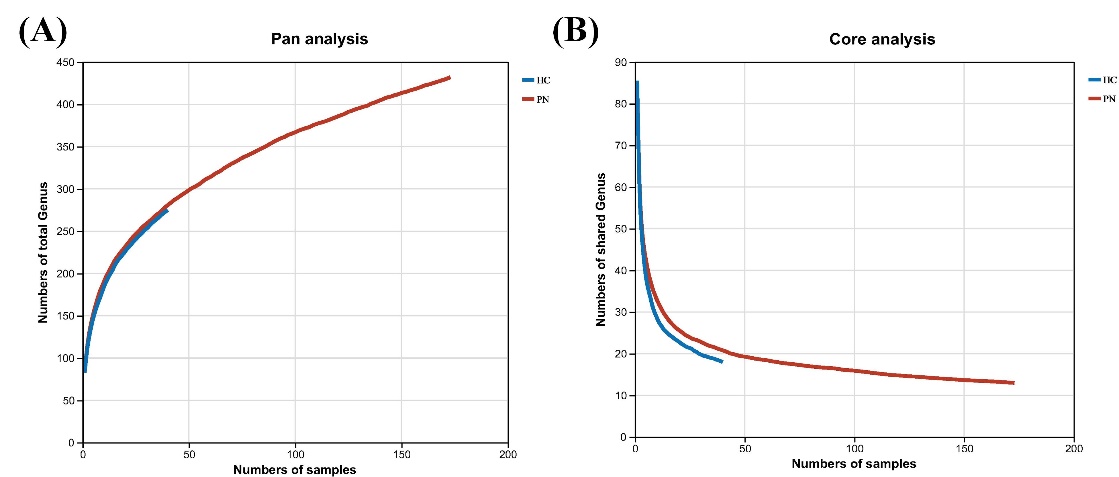


**Supplementary Figure 1 | (A)** Pan analysis to observe the changes in the total number of species in the HC and PN groups as the number of samples increases. **(B)** Core analysis to observe the changes in core species numbers of the HC and PN groups as the sample size increases as the number of samples increases. PN, pulmonary nodule; HC, healthy control.
