# Supplementary material for: Saliva‑microbiome‑derived signatures: expected to become a potential biomarker for pulmonary nodules (MCEPN-1)
Source: BMC Microbiol. 2024 Apr 20;24:132. doi: 10.1186/s12866-024-03280-x (PMC11031921; doi:10.1186/s12866-024-03280-x)
Supplement: Supplementary file 4 — Supplementary Material 4 [file 12866_2024_3280_MOESM4_ESM.docx]

**Supplementary Figure 2**

**Supplementary Figure 2 |** Rank abundance curves of the HC and PN groups. The x-axis is the abundance rank, and the y-axis is the relative abundance. PN, pulmonary nodule; HC, healthy control.
