# Supplementary material for: Saliva‑microbiome‑derived signatures: expected to become a potential biomarker for pulmonary nodules (MCEPN-1)
Source: BMC Microbiol. 2024 Apr 20;24:132. doi: 10.1186/s12866-024-03280-x (PMC11031921; doi:10.1186/s12866-024-03280-x)
Supplement: Supplementary file 5 — Supplementary Material 5 [file 12866_2024_3280_MOESM5_ESM.docx]

**Supplementary Figure 3**


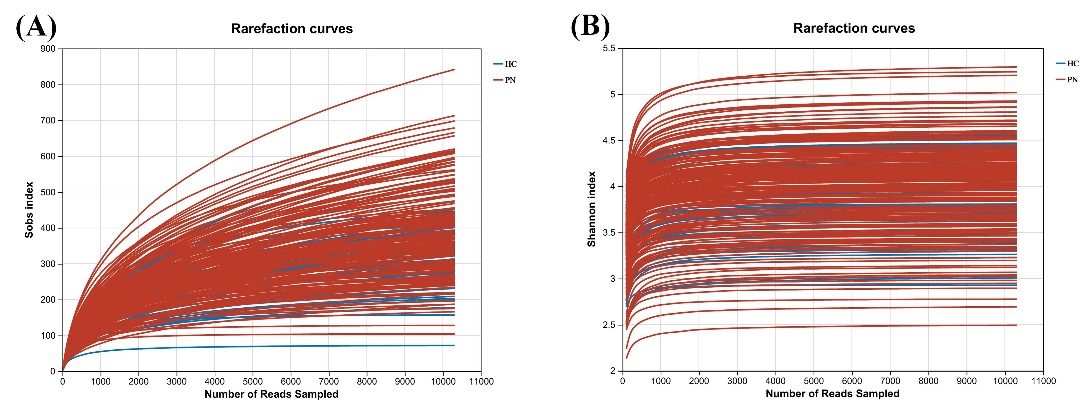


**Supplementary Figure 3 | (A)** Rarefaction curves of Sobs index in the PN and HC groups. **(B)** Rarefaction curves of Shannon index in the PN and HC groups. Red and blue curves represent the amplified sequence variant analysis of the PN and HC groups. PN, pulmonary nodule; HC, healthy control.
