# Supplementary material for: Saliva‑microbiome‑derived signatures: expected to become a potential biomarker for pulmonary nodules (MCEPN-1)
Source: BMC Microbiol. 2024 Apr 20;24:132. doi: 10.1186/s12866-024-03280-x (PMC11031921; doi:10.1186/s12866-024-03280-x)
Supplement: Supplementary file 6 — Supplementary Material 6 [file 12866_2024_3280_MOESM6_ESM.docx]

**Supplementary Figure 4**


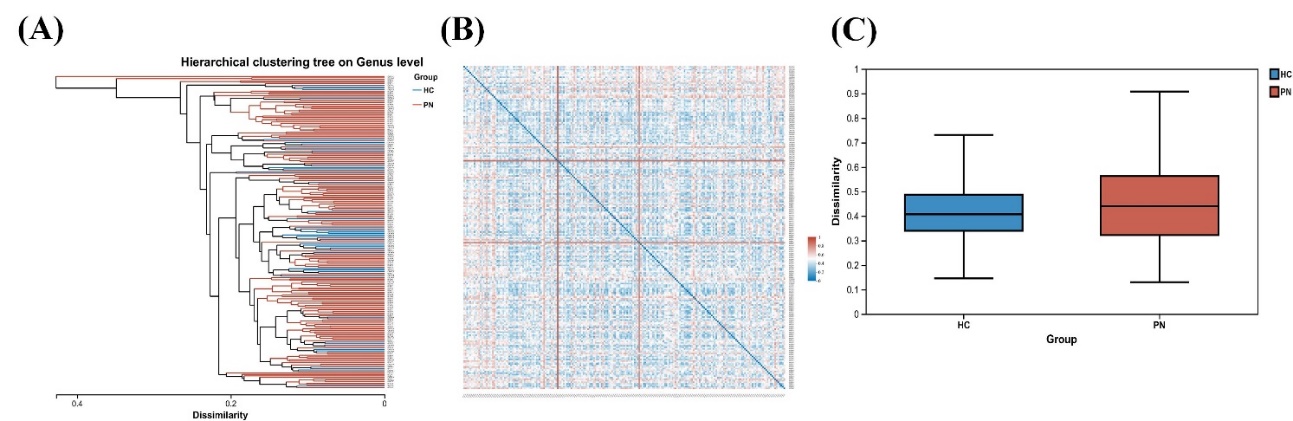


**Supplementary Figure 4 |** Hierarchical cluster analysis based on the Bray-Curtis algorithm of the two groups. (**A**) Hierarchical clustering tree of microbes on genus level. (**B**) Heatmap of hierarchical clustering of microbes on genus level. (**C**) Distance boxplot of hierarchical clustering of microbes on genus level of the two groups. PN, pulmonary nodule; HC, healthy control.
